# Distinct patterns of cortical activation and functional connectivity in children with high-functioning autism during a verbal fluency task: a comparative fNIRS study

**DOI:** 10.3389/fnins.2026.1736415

**Published:** 2026-04-01

**Authors:** Bin Yu, Jian-Min Lv, Ge Lei, Jing-Li Wu, Ding-Xu Li, Xuan-Yue Song, Xi-Ning He, Na Zhao, Qian Shu, Hong-Xia Li

**Affiliations:** 1 Pediatric Medical Rehabilitation Center, Shaanxi Provincial Rehabilitation Hospital, Xi'an, Shaanxi, China; 2 Rehabilitation Science Institute, Shaanxi Provincial Rehabilitation Hospital, Xi'an, Shaanxi, China

**Keywords:** cortical activation, functional connectivity, functional near-infrared spectroscopy, high-functioning autism, language production, verbal fluency task

## Abstract

**Objective:**

Children with High-Functioning Autism (HFA) often show marked deficits in executive functioning, particularly during verbal fluency tasks (VFTs). These behavioral impairments are believed to stem from neurophysiological abnormalities in the prefrontal cortex (PFC) functioning, characterized by atypical activation patterns and disrupted functional connectivity. This study utilized functional near-infrared spectroscopy (fNIRS) to investigate hemodynamic responses and connectivity metrics during VFT performance. By comparing children with HFA to age-matched typically developing (TD) controls, this study aimed to clarify the neural mechanisms underlying the executive control of language production in HFA.

**Methods:**

The sample included 29 children who met diagnostic criteria for HFA and 26 TD controls. All participants had a Full-Scale Intelligence Quotient of 70 or higher and were matched for age and cognitive ability. During a standardized phonemic VFT, cortical hemodynamics were continuously monitored using a 19-channel fNIRS system, with analyses focusing on changes in oxygenated hemoglobin concentration within PFC regions.

**Results:**

Compared with TD controls, children with HFA exhibited reduced cortical activation across multiple prefrontal regions, including channels 1 (*t* = −2.975, *p* = 0.017), 2 (*t* = −4.320, *p* = 0.001), 3 (*t* = −3.381, *p* = 0.012), 9 (*t* = −3.127, *p* = 0.014), and 19 (*t* = −3.279, *p* = 0.012). These regions correspond anatomically to the inferior prefrontal gyrus, frontopolar cortex, and dorsolateral PFC. Functional connectivity analyses demonstrated significantly reduced interregional coupling in the HFA group (*p* < 0.001), with mean connectivity values of 0.512 (SD = 0.076) compared with 0.566 (SD = 0.069) in TD participants. Furthermore, Oxy-Hb changes in prefrontal channels 1 (*r* = −0.424, *p* = 0.022), 2 (*r* = −0.432, *p* = 0.019), and 3 (*r* = −0.394, *p* = 0.034) were negatively correlated with Social Responsiveness Scale total scores, indicating that weaker prefrontal activation was associated with greater social impairment.

**Conclusion:**

The results reveal distinct cortical activation and functional connectivity alterations in children with HFA during VFTs. These findings support the hypothesis that disrupted interregional brain coordination underlies executive difficulties in language production in HFA children, who exhibit reduced PFC activation and weaker interregional functional connectivity during the VFT.

## Introduction

Autism Spectrum Disorder (ASD) is a complex neurodevelopmental condition characterized by persistent deficits in social communication and interaction, along with restricted and repetitive patterns of behavior, interests, or activities (Meza et al., 2025; Singhi and Malhi, 2023). Recent epidemiological studies report an increasing global prevalence of ASD. For instance, the latest surveillance data from the United States indicates a prevalence of 1 in 31 children aged 8 years, although reported rates vary across regions due to differences in diagnostic practices and screening comprehensiveness (Shaw et al., 2025). This high and potentially increasing prevalence underscores the substantial public health significance and socioeconomic burden for affected individuals, families, and healthcare systems (Wormwood et al., 2023; Hirota and King, 2023; Salari et al., 2022). This rising prevalence highlights the urgent need for research aimed at elucidating the neurobiological underpinnings of ASD and developing effective interventions (Clothier and Absoud, 2021; Dunalska et al., 2021; Jiang et al., 2022; Kereszturi, 2023).

Many children with ASD demonstrate average or above-average intellectual functioning, as measured by the Full-Scale Intelligence Quotient (FSIQ ≥ 70) yet continue to exhibit marked impairments in social adaptation relative to their cognitive capacity (Audras-Torrent et al., 2021). Among these individuals, difficulties in effective communication, particularly in the fluid and goal-directed generation of language, are particularly prominent, as reflected in challenges during structured expressive tasks or in assessments of verbal fluency (Larson et al., 2021; Takayanagi et al., 2022). Given the fundamental role of efficient language production in social interaction and adaptive functioning, interventions targeting this domain may substantially enhance social outcomes in individuals with High-Functioning Autism (HFA) (Panerai et al., 2014; Kenworthy et al., 2010). However, the neurocognitive mechanisms contributing to these expressive language and communication deficits remain poorly understood (Baranova et al., 2021).

The verbal fluency task (VFT) is an established paradigm for examining the executive control processes underlying language production (Fedorenko and Blank, 2020; Vigneau et al., 2006; Andersson, 2010; Ohtani et al., 2021; Amunts et al., 2020; Chami et al., 2022; Hirshorn and Thompson-Schill, 2006; Heim et al., 2009; Hegarty et al., 2020; Wang Y. et al., 2025). Successful language production requires not only lexical access but also domain-general executive functions such as strategic search, cognitive flexibility, and inhibitory control, which are primarily supported by the prefrontal cortex (PFC). The VFT, by requiring participants to generate words rapidly under specific constraints, directly probes this interface between language production and prefrontal executive systems. Its performance thus critically depends on the integrity of prefrontal control regions and efficient functional connectivity within the associated neural networks. Investigating neural activation during VFT performance in children with HFA may therefore offer valuable insight into the neural mechanisms underlying their characteristic difficulties in the executive aspects of communication (Fedorenko and Blank, 2020; Vigneau et al., 2006; Andersson, 2010; Ohtani et al., 2021; Amunts et al., 2020; Chami et al., 2022; Hirshorn and Thompson-Schill, 2006; Heim et al., 2009; Hegarty et al., 2020; Wang Y. et al., 2025).

Although neuroimaging studies have consistently documented atypical neural activation and disrupted functional connectivity during executive function tasks in individuals with HFA (Jiang et al., 2022; Lamanna and Meldolesi, 2024; Velarde and Cárdenas, 2022; Wang et al., 2023), three key knowledge gaps remain for high-functioning populations. First, the specific nature of cortical activation alterations in core brain regions during VFT performance remains unclear. It is not yet known whether these changes reflect hyperactivation, hypoactivation, or atypical activation patterns (Loth et al., 2017). Second, prior studies examining task-modulated functional connectivity have reported inconsistent results, often constrained by methodological limitations (Hutchison et al., 2013; Xiao et al., 2025). Third, few investigations have simultaneously explored both localized neural responses and distributed network connectivity during task execution, despite the theoretical importance of their interaction for understanding executive dysfunction in HFA (Uddin et al., 2013). Importantly, the clinical relevance of these neural abnormalities is still uncertain, particularly regarding their association with core social impairments. Clarifying this relationship could provide critical mechanistic insights and contribute to the identification of potential biomarkers.

Functional near-infrared spectroscopy (fNIRS) offers distinct methodological advantages for developmental neuroimaging studies, particularly its tolerance to head movement and its compatibility with naturalistic testing conditions that permit verbal responses (Brigadoi et al., 2014; Yücel et al., 2021; Gunasekara et al., 2022; Pinti et al., 2020). Critically, its established utility and growing application in studying neurodevelopmental disorders, including autism spectrum disorder, are well documented in recent comprehensive reviews (Wang J. et al., 2025). These features make fNIRS particularly well suited for examining cortical activation during active language production tasks such as the VFT in pediatric populations, including children with HFA (Chien et al., 2025; Cetik et al., 2025). While fNIRS is well-suited for such pediatric and ecologically-valid paradigms, it is important to consider its limitations alongside its strengths, such as its relatively lower spatial resolution compared to fMRI and sensitivity to certain physiological artifacts (Pliatsikas, 2024).

Building upon these technical strengths, the present study used fNIRS to address three key research objectives in a developmentally homogeneous pre-adolescent cohort (6–10 years old). The first objective was to determine whether high-functioning children with HFA (FSIQ ≥ 70) exhibit distinct cortical activation patterns in VFT-related brain regions compared with age- and FSIQ-matched typically developing (TD) controls. The second objective was to examine potential group differences in the strength of functional connectivity within prefrontal cortical networks. The third objective was to explore whether abnormal neural activation patterns in the HFA group are associated with the severity of clinical symptoms.

By concurrently analyzing regional brain activation and interregional functional connectivity during VFT performance, alongside clinical symptom assessment, this study seeks to clarify the neural correlates of the executive control of language production and executive function impairments in HFA. Identifying significant associations between specific cortical abnormalities (e.g., prefrontal hypoactivation) andelevated Social Responsiveness Scale (SRS) scores would provide crucial evidence of brain-behavior relationships. Such findings would not only advance the mechanistic understanding of HFA pathophysiology but also inform the identification of potential neural targets for therapeutic intervention.

## Materials and methods

### Subjects

Participants met the following inclusion criteria: pre-adolescent children aged 6–10 years old (a strictly defined pre-adolescent developmental period with no pubertal neuromaturational changes) who had received a formal HFA diagnosis according to DSM-5 criteria and completed the Autism Diagnostic Observation Schedule-2nd Edition assessment, with clinically stable symptoms (First, 2013; Yu et al., 2024). This age range was selected to eliminate the interference of pubertal neurodevelopmental changes, ensure adequate cognitive capacity for task performance, and focus on the core childhood period of prefrontal cortex development. Exclusion criteria included: (1) intellectual disability (FSIQ < 70); (2) comorbid Attention-Deficit/Hyperactivity Disorder; or (3) symptoms attributable to genetic or metabolic disorders. All participants underwent comprehensive neuropsychological evaluation, including the Wechsler Intelligence Scale for Children-IV for cognitive assessment and standardized HFA symptom measures. Based on diagnostic status and cognitive functioning (FSIQ ≥ 70), participants were stratified into two matched groups: an HFA group (*n* = 29) and a TD control group (*n* = 26). All assessments were administered by doctoral-level clinical psychologists to ensure diagnostic reliability.

The final cohort comprised 55 carefully characterized pre-adolescent participants (6–10 years old) recruited through Shaanxi Provincial Rehabilitation Hospital between October 2024 and Febrary 2026. The study protocol was approved by the Institutional Review Board of Shaanxi Provincial Rehabilitation Hospital (Approval Number: SXKF-2024062), and written informed consent was obtained from the parents or legal guardians of all participants prior to enrollment.

A *post hoc* power analysis was performed using G Power 3.1.9.7 to verify the statistical adequacy of the sample size. For two-tailed independent t-tests with *α* = 0.05 and Cohen’s d = 0.8 (based on the primary prefrontal channel activation differences), the achieved power was 0.83 for the sample of HFA = 29 and TD = 26, which fully meets the statistical power requirements for detecting large effect sizes in neuroimaging studies of pediatric HFA populations.

### VFT

VFT was administered during daytime sessions following a standardized procedure. The paradigm consisted of three sequential phases: (1) a 30-s pre-task baseline period in which participants engaged in simple numerical counting (repetitive of numbers 1–5); (2) a 60-s active task phase requiring generative language production; and (3) a 60-s post-task recovery baseline repeating the counting procedure. During the active task phase, participants generated two-word phrases using three target Chinese characters (e.g., 白, 北, and 大, meaning “white,” “north,” and “big,” respectively). This set of characters is a well-established phonemic fluency stimulus in Mandarin fNIRS research, having been used in studies across various clinical and cognitive domains (Zhang et al., 2024; Bian et al., 2025). The character sets were systematically rotated every 20 s to maintain cognitive engagement and minimize response latency.

### fNIRS measurement

**Figure 1:**
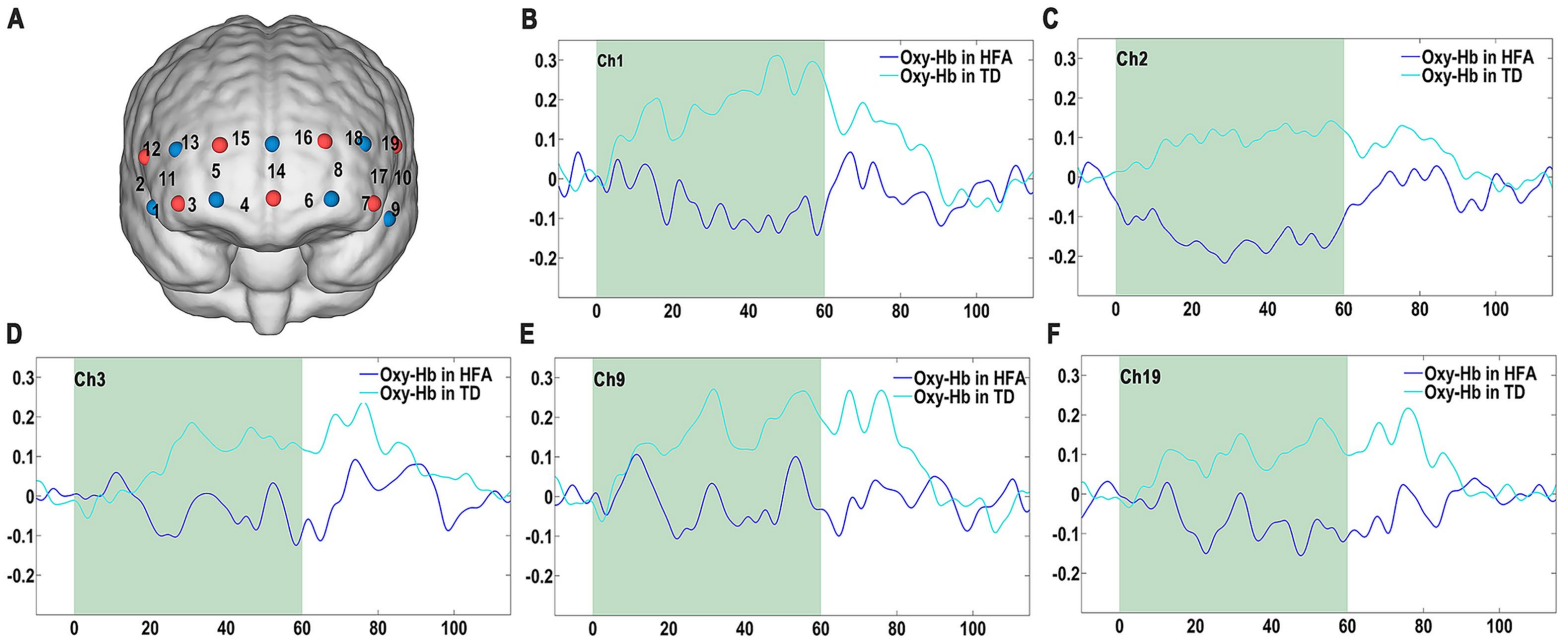
**(A)** Positions of the 19 fNIRS channels. **(B–F)** Time courses of mean Oxy-Hb concentration changes in channels 1, 2, 3, 9, and 19 during the VFT in the HFA and TD groups.

Participants were seated comfortably in a quiet room. Hemoglobin responses were measured using a multi-channel near-infrared optical imaging system (NirSmart-6000A equipment, Danyang Huichuang Medical Equipment Co., Ltd., China). The sampling rate was 11 Hz, and light wavelengths were 730 nm and 850 nm, with 808 nm used as an isotopic reference. A total of 14 SD probes, consisting of 7 sources and 7 detectors, were arranged with a fixed 3-cm source-detector separation distance to cover bilateral prefrontal cortex (PFC), forming 19 fNIRS channels. Based on this distance, the maximum probing depth for the hemodynamic signal is estimated to be approximately 1.5 cm from the scalp surface, which adequately encompasses the cortical gray matter of the prefrontal regions under investigation. Channel localization was as follows: channels 1, 2, and 9 corresponded to Inferior prefrontal gyrus (IFG); channels 3–8, 11, and 13–18 covered the frontopolar cortex (FPC); channel 10 corresponded to the pars triangularis Broca’s area; and channels 12 and 19 represented the dorsolateral prefrontal cortex (DLPFC) (see Figure 1A). Channel anatomical localization was estimated by co-registering the optode positions (placed according to the 10–20 system) to the Montreal Neurological Institute (MNI) standard brain space using the NirSpark platform (Liu et al., 2022). The probe array was positioned to provide comprehensive coverage of prefrontal executive regions hypothesized to be central to VFT performance. Due to the finite number of channels and the primary focus on prefrontal contributions to executive aspects of language production, the array did not extend coverage to posterior temporal regions such as Wernicke’s area. Most inpatients completed the fNIRS evaluation within 3 days of hospital admission.

### Data processing and analysis

The fNIRS data preprocessing was performed using the NirSpark analytical platform (HuiChuang, China). Motion artifacts were corrected using a two-stage procedure: identification of signal deviations via moving standard deviation analysis, followed by signal reconstruction using cubic spline interpolation. Physiological noise was reduced through bandpass filtering (0.01–0.20 Hz) to remove cardiopulmonary and low-frequency artifacts. Optical density data were subsequently converted to hemoglobin concentration using the modified Beer–Lambert law. Oxygenated hemoglobin (Oxy-Hb) was selected as the primary indicator because of its superior signal reliability (Strangman et al., 2002). Task-related hemodynamic responses were analyzed within a 125-s time window, including a 10-s pre-task baseline, a 60-s active VFT period, and a 55-s post-task recovery phase. The active phase served as the main analysis period for quantifying mean Oxy-Hb changes, with baseline correction performed using linear regression. Final hemodynamic response waveforms were averaged across all 19 channels for each group.

Data visualization was performed using NirSpark, Origin 2021, and Adobe Photoshop 2025. The Shapiro–Wilk test was applied to assess data normality. Group differences in demographic variables were analyzed using either unpaired t-tests for continuous variables or chi-square tests for categorical variables. Between-group differences in Oxy-Hb changes, reflecting neural activation patterns, were evaluated using t-tests comparing HFA and TD groups. Functional connectivity was quantified by calculating Spearman’s correlation coefficients for all possible channel-to-channel pairs based on their time series data. Statistical significance was defined as *p* < 0.05 (two-tailed). Pearson correlation analyses were conducted to investigate associations between regional brain activation changes and clinical measures within each group. All statistical analyses were performed using SPSS 27.0 (IBM Corp., Armonk, NY, USA).

## Results

### Basic characteristics of the participants

Table 1 summarizes the demographic and cognitive profiles of the participants according to diagnosis and FSIQ. The HFA group (FSIQ ≥ 70) included 27 males and 2 females, with a mean age of 7.97 years (SD = 1.32). The TD group (FSIQ ≥ 70) comprised 23 males and 3 females, with a mean age of 8.27 years (SD = 1.37). Intergroup comparisons revealed no statistically significant differences in age (*t* = −0.835, *p* = 0.407), gender distribution (*χ*^2^ = 0.357, *p* = 0.550), delivery mode (*χ*^2^ = 1.401, *p* = 0.237), parental care status (*χ*^2^ = 2.648, *p* = 0.104), or only-child status (χ^2^ = 0.266, *p* = 0.606). In cognitive assessments, the HFA group exhibited significantly lower scores on the VCI (Verbal Comprehension Index) (*p* < 0.01) compared to the TD group. No significant differences were observed in the Perceptual Reasoning Index (PRI; *p* = 0.502) or FSIQ (*p* = 0.157). The mean scores in the HFA group were 73.83 (SD = 19.16) for VCI, 96.90 (SD = 17.52) for PRI, and 81.06 (SD = 11.77) for FSIQ. The corresponding values for the TD group were 86.96 (SD = 15.15) for VCI, 93.58 (SD = 18.90) for PRI, and 86.54 (SD = 16.34) for FSIQ. On the SRS, the HFA group scored significantly higher than the TD group (*t* = 17.371, *p* < 0.001), with mean scores of 95.28 (SD = 17.86) and 33.85 (SD = 6.25), respectively.

**Table 1:** Demographic and clinical characteristics (mean ± SD).

Variable	HFA (*n* = 29)	TD (*n* = 26)	*t*/Chi-square value	*p*
Demographics				
Age (years)	7.97 ± 1.32	8.27 ± 1.37	−0.835	0.407
Gender (male/female)	27/2	23/3	0.357	0.550
Delivery mode (yes/no)	18/11	12/14	1.401	0.237
Parental care (yes/no)	15/14	19/7	1.899	0.168
Being an only child (yes/no)	17/12	17/9	0.266	0.606
Clinical characteristics				
Wechsler Children’s Intelligence Test-IV				
Verbal comprehension index (VCI)	73.83 ± 19.16	86.96 ± 15.15	−2.834	**0.007**
Perceptual reasoning index (PRI)	96.90 ± 17.52	93.58 ± 18.90	0.673	0.504
Full-scale intelligence quotient (FSIQ)	81.06 ± 11.77	86.54 ± 16.34	−1.410	0.165
Social responsiveness scale (SRS)	95.28 ± 17.86	33.85 ± 6.25	16.617	**<0.001**
VFT behavioral performance				
Correct words/minute	3.41 ± 1.18	3.92 ± 1.02	−1.718	0.092

Bold values indicate statistical significance (*p* < 0.05).

### Behavioral performance on the VFT

The behavioral data extracted during the fNIRS recording provided a direct measure of task performance. While both groups engaged in the phonemic fluency task, children with HFA showed a numerical trend toward reduced verbal output (Table 1). Specifically, the mean number of correct words generated per minute was lower in the HFA group (3.41 ± 1.18) than in the TD group (3.92 ± 1.02). An independent samples t-test indicated that this difference was not statistically significant (*t* = −1.718, *p* = 0.094). This pattern of marginally impaired behavioral performance aligns with the observed neural differences, providing a behavioral context for the cortical activation and connectivity findings reported below.

### Mean Oxy-Hb changes during the VFT

fNIRS was used to assess hemodynamic responses in 19 predefined cortical regions during the VFT, with channel locations illustrated in Figure 1A. Comparative analysis of grand-averaged Oxy-Hb waveforms (Figures 1B–F; Supplmentary Figure S1) revealed significant between-group differences in temporal activation profiles. Specifically, children with HFA exhibited atypical hemodynamic response patterns, including altered slope progression and variable peak amplitudes across multiple channels, suggesting potential disruptions in neurovascular coupling during cognitive processing.

In contrast, the TD group exhibited characteristic hemodynamic responses, with Oxy-Hb concentrations progressively increasing during task execution and returning gradually to baseline during the post-task period (Figures 1B–F; Supplmentary Figure S1). Quantitative analysis confirmed significantly reduced Oxy-Hb activation in the HFA group across five prefrontal regions (Ch1, 2, 3, 9, and 19), corresponding anatomically to the IFG, FPC, and DLPFC (Figures 1B–F). These differences in cortical activation patterns persisted throughout the entire task duration.

**Figure 2:**
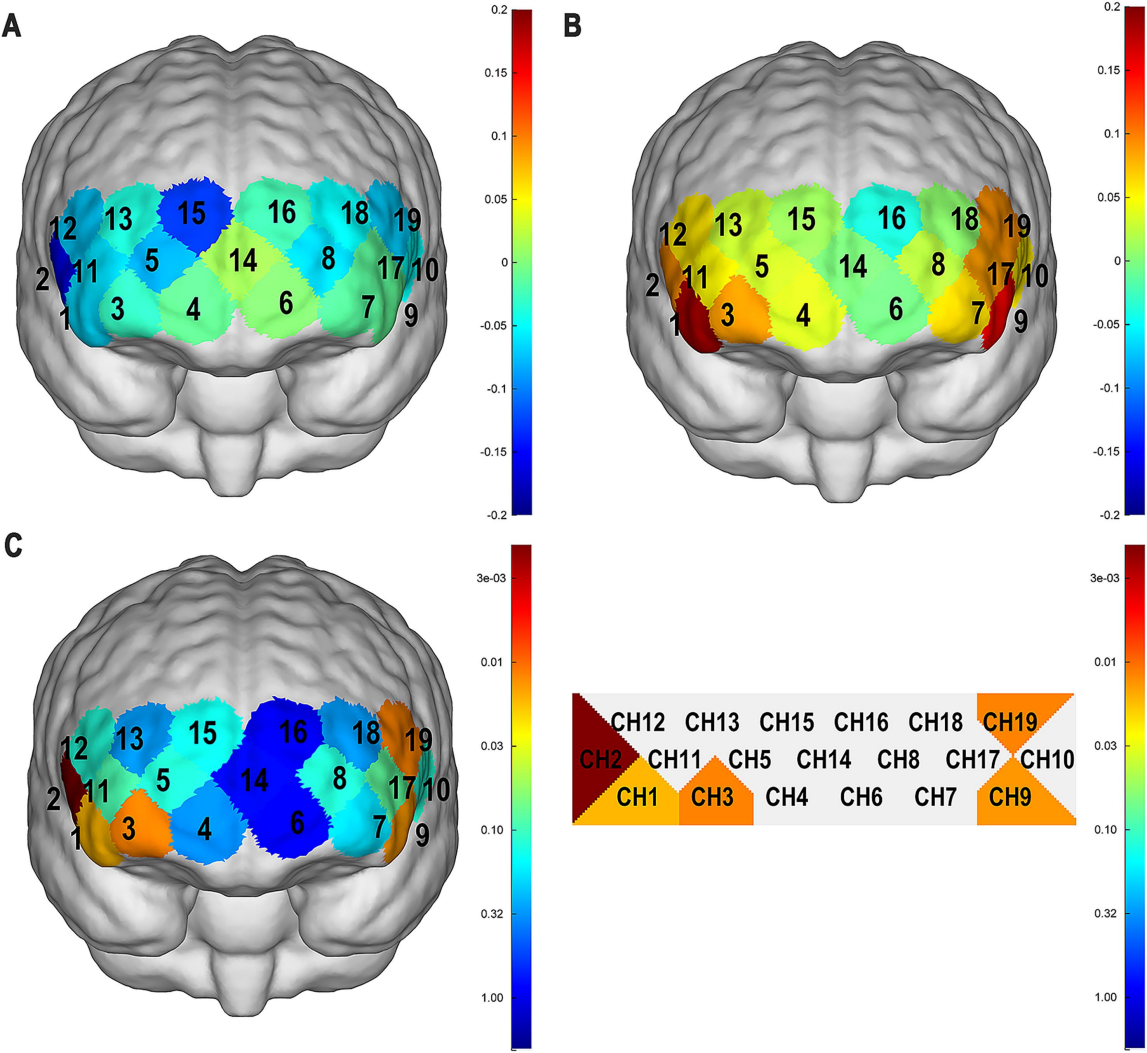
**(A)** Mean Oxy-Hb changes in each channel of the ASD group. **(B)** Mean Oxy-Hb change in each channel of the TD group. **(C)** *p*-values for channel activation levels in the HFA group relative to the TD group.

Statistical analysis of mean Oxy-Hb changes revealed significant between-group differences in PFC activation patterns (Figure 2; Table 2). Children with HFA showed significantly attenuated hemodynamic responses in the five prefrontal regions during task performance, with reductions observed in channel 1 (*t* = −2.975, *p* = 0.017), channel 2 (*t* = −4.320, *p* = 0.001), channel 3 (*t* = −3.381, *p* = 0.012), channel 9 (*t* = −3.127, *p =* 0.014), and channel 19 (*t* = −3.279, *p* = 0.012). These findings quantitatively support the distinct waveform alterations identified in the initial analysis.

**Table 2:** Differences in oxygenated hemoglobin concentration during two sets of channel-based task states.

Channel	S-D	HFA	TD	*t*	FDR-corrected *p*
1	S2-D2	−0.0655	0.1827	−2.975	**0.017**
2	S2-D7	−0.1525	0.0927	−4.320	**0.001**
3	S3-D2	−0.0296	0.0872	−3.381	**0.012**
4	S3-D3	−0.0105	0.0349	−1.103	0.327
5	S3-D8	−0.0791	0.0315	−1.948	0.120
6	S4-D3	0.0031	−0.0044	0.123	0.903
7	S4-D4	−0.0151	0.0617	−1.550	0.186
8	S4-D9	−0.0620	0.0228	−1.852	0.120
9	S5-D4	−0.0117	0.1484	−3.127	**0.014**
10	S5-D10	−0.0491	0.0570	−2.020	0.115
11	S8-D2	−0.0610	0.0510	−2.186	0.090
12	S8-D7	−0.0705	0.0587	−1.861	0.120
13	S8-D8	−0.0381	0.0292	−1.249	0.288
14	S9-D3	0.0134	−0.0016	0.356	0.808
15	S9-D8	−0.1276	0.0112	−1.743	0.138
16	S9-D9	−00224	−0.0379	0.188	0.899
17	S10-D4	−0.0135	0.0874	−2.306	0.079
18	S10-D9	−0.0492	0.0047	−1.222	0.288
19	S10-D10	−0.0691	0.0907	−3.279	**0.012**

Bold values indicate statistical significance (*p* < 0.05).

### Functional connectivity

**Figure 3:**
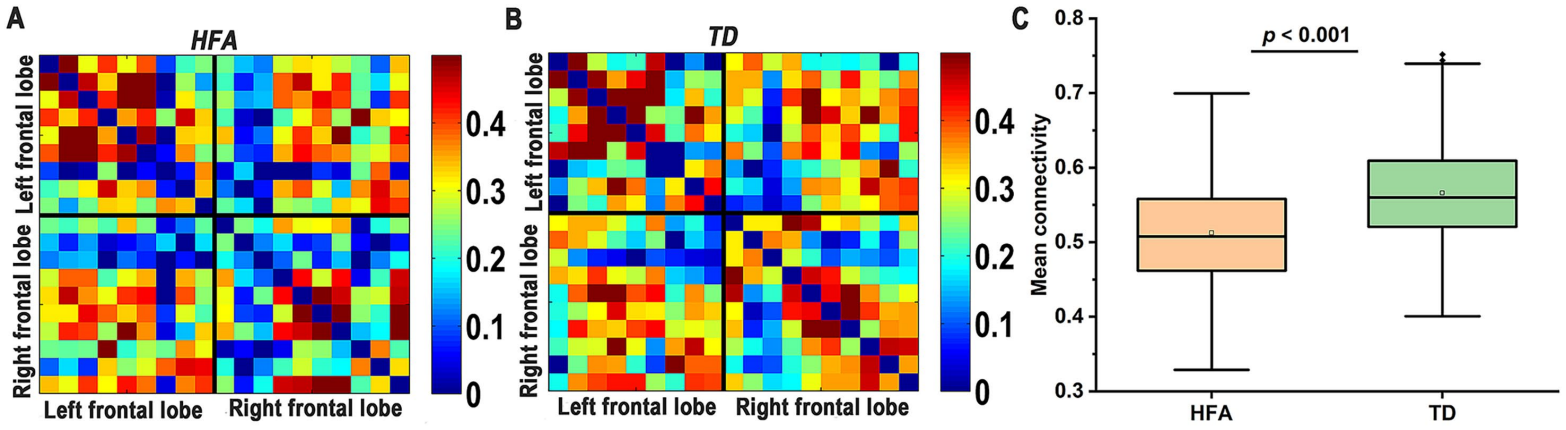
**(A,B)** Connectivity between the hemodynamic responses across the 19 channels. Left frontal lobe: channels 6–10, 16–19; Right frontal lobe: channels 1–5, 11–15. **(C)** Mean channel-to-channel connectivity strength was lower in the HFA group than in the TD group.

Functional connectivity analysis generated 19 × 19 correlation matrices for each experimental group (HFA: Figure 3A; TD: Figure 3B; Supplementary Tables S1). Our primary analysis focused on the global mean connectivity strength, computed by averaging correlation coefficients across all unique channel pairs. This measure revealed a significant reduction in children with HFA (Mean = 0.512, SD = 0.076) relative to TD controls (Mean = 0.566, SD = 0.069), with this group difference reaching statistical significance (*p* < 0.001, Figure 3C). These results indicate impaired neural network integration in children with HFA.

### Correlations with clinical characteristics

**Figure 4:**
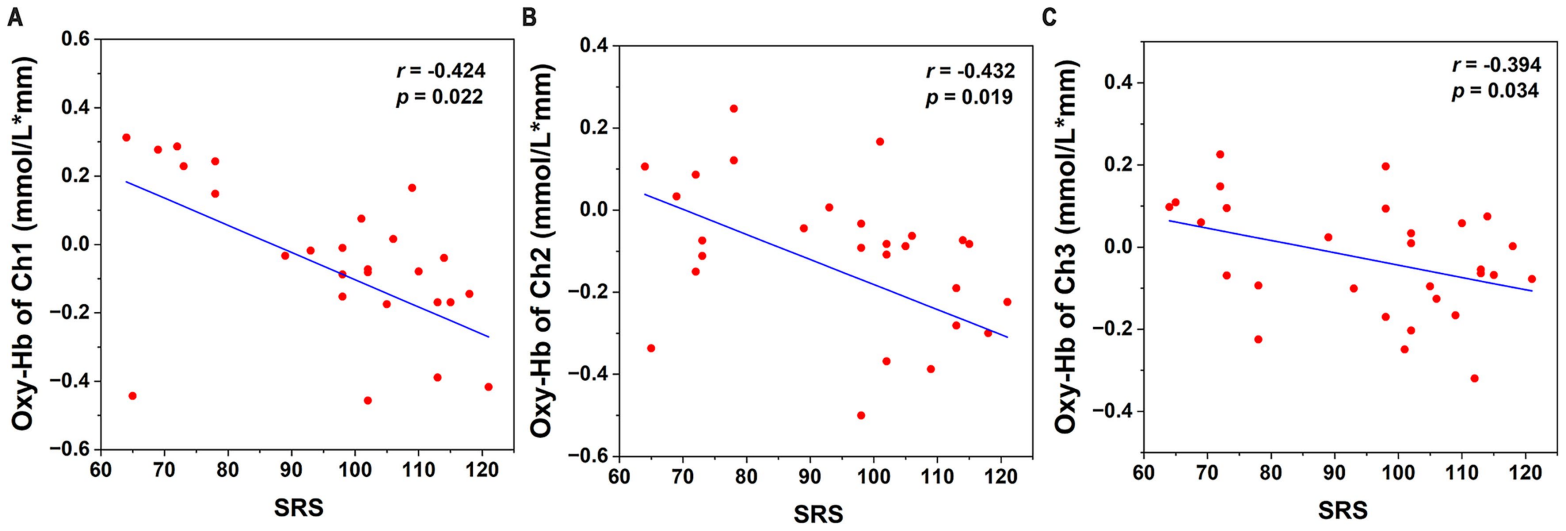
Correlation between SRS scores and mean Oxy-Hb concentration changes. **(A)** Channel 1, **(B)** Channel 2, **(C)** Channel 3.

Correlation analyses demonstrated significant inverse relationships between SRS scores and mean Oxy-Hb changes in prefrontal channels 1 (*r* = −0.424, *p* = 0.022), 2 (*r* = −0.432, *p* = 0.019), and 3 (*r* = −0.394, *p* = 0.034) (Table 3; Figure 4). No significant associations were found between hemodynamic responses and cognitive measures (VCI, PRI, or FSIQ) or demographic variables, including age, sex, parental status, or singleton status. Complete statistical outputs are provided in Supplementary Table S2.

**Table 3:** Associations between channel Oxy-Hb changes and clinical characteristics in the HFA group.

Channel	Verbal comprehension index (VCI)		Perceptual reasoning index (PRI)		Full-scale intelligence quotient (FSIQ)		Social responsiveness scale (SRS)	
	*r*	*p*	*r*	*p*	*r*	*p*	*r*	*p*
1	−0.105	0.589	−0.180	0.350	−0.317	0.094	−0.424	**0.022**
2	0.101	0.609	−0.044	0.821	−0.065	0.738	−0.432	**0.019**
3	−0.110	0.571	0.226	0.238	−0.009	0.964	−0.394	**0.034**
4	−0.203	0.291	0.361	0.054	0.046	0.812	−0.145	0.454
5	0.121	0.532	−0.148	0.445	−0.070	0.717	−0.295	0.120
6	−0.253	0.186	0.245	0.199	−0.080	0.679	0.003	0.987
7	−0.165	0.393	0.118	0.544	−0.160	0.407	−0.160	0.406
8	−0.128	0.508	0.108	0.576	0.043	0.826	−0.112	0.562
9	0.200	0.299	−0.065	0.738	−0.052	0.789	−0.007	0.971
10	0.030	0.877	−0.032	0.869	−0.039	0.840	0.097	0.617
11	0.138	0.474	−0.143	0.459	−0.128	0.509	0.156	0.420
12	−0.228	0.234	0.067	0.731	−0.291	0.125	0.201	0.296
13	−0.075	0.697	−0.093	0.631	−0.262	0.170	−0.019	0.923
14	0.182	0.345	0.082	0.672	0.092	0.635	0.098	0.611
15	0.203	0.291	−0.149	0.440	−0.003	0.989	0.128	0.508
16	0.251	0.188	−0.278	0.145	−0.078	0.688	0.106	0.584
17	−0.026	0.893	−0.130	0.500	−0.233	0.225	−0.116	0.551
18	0.034	0.859	−0.005	0.980	−0.001	0.996	0.087	0.654
19	0.049	0.801	0.094	0.629	0.015	0.938	0.017	0.932

Bold values indicate statistical significance (*p* < 0.05).

## Discussion

This study employed fNIRS to examine cortical activation patterns in pre-adolescent children (6–10 years old) with HFA and TD controls during a VFT. The findings revealed significant group differences in PFC activation, which corresponded to variations in the executive demands of verbal production among participants.

Comparative analysis of children with FSIQ ≥ 70 demonstrated significantly reduced prefrontal activation in the HFA group relative to TD controls. Specifically, attenuated hemodynamic responses were observed in channels 1, 2, 9 (IFG), 3 (FPC), and 19 (DLPFC). These three cortical regions function as a tightly coordinated neural hub responsible for high-order cognitive integration. The DLPFC serves as a principal executive center mediating reasoning, decision-making, and working memory maintenance. he IFG supports articulated speech production and contributes to behavioral regulation by inhibiting prepotent responses. The FPC, located at the most anterior portion of the PFC, supports multitasking and prospective cognition, enabling future-oriented planning and mental simulation. Together, these regions provide complementary computational functions that underline the coordination of complex cognitive operations. The present results are consistent with prior evidence demonstrating the critical role of the IFG in language processing and executive function (Friederici, 2011; Fujii et al., 2016; Specht, 2014), suggesting that altered activation in this region may underlie core HFA symptoms. Moreover, the observed pattern of prefrontal hypoactivation is consistent with previous reports in children with HFA during cognitive tasks (Just et al., 2012; Solomon et al., 2009; Dichter et al., 2009).

Functional connectivity analyses further revealed significantly reduced global neural network integration (mean connectivity strength) in children with HFA compared to FSIQ-matched TD controls. Quantitative measures indicated weaker interregional connectivity in the HFA group during VFT, particularly in networks supporting language and executive function. The observed pattern of uncorrected differences at the channel-pair level suggests a spatially non-uniform profile of this hypoconnectivity, which warrants future investigation in larger samples. These findings align with previous reports of atypical connectivity patterns in children with HFA and further demonstrate that such abnormalities persist even in cognitively able individuals (Müller et al., 2011; Ma et al., 2022; Nomi and Uddin, 2015; Yao et al., 2021).

Neurofunctional correlations revealed significant negative associations between SRS scores and PFC, with reduced Oxy-Hb changes observed in the IFG (channels 1–2) and FPC (channel 3) in childre with HFA. The HFA group also showed significantly lower verbal comprehension scores than TD controls. These findings suggest that domain-specific neural dysfunction within language-related prefrontal regions contributes to both social communication difficulties and verbal deficits in HFA. Notably, no significant relationships were found between hemodynamic activity and perceptual reasoning or FSIQ, indicating that the observed abnormalities are specifically linked to language processing rather than general cognitive ability. Demographic factors such as age, gender, and family structure showed no significant effects on activation patterns.

Our study highlights the relationship between prefrontal function, the executive control of language production, and autism -related symptoms in children with HFA. Activity within the IFG, FPC, and DLPFC emerged as potential neural markers, with stronger activation in these regions associated with better language performance and reduced autism symptom severity.

These present findings also demonstrate that fNIRS is a valuable tool for assessing language-related brain function in children with HFA. These results provide important neurobiological insight into the mechanisms underlying language dysfunction in HFA and demonstrate potential clinical applications for individualized intervention through modulation of prefrontal activity.

For exploratory validation of the robustness of our primary findings, we additionally conducted analyses on the original wider age cohort (6–13 years old). It should be noted that the current 6–10 years pre-adolescent sample is an adjusted version of the original cohort, retaining most of the original 6–10 years participants with newly recruited subjects added and all adolescent participants (> 10 years old) excluded. The overall patterns of prefrontal cortical Oxy-Hb activation differences, inter-channel functional connectivity characteristics, and the associations between neural indicators and clinical symptoms in the 6–13 years cohort were highly consistent with the primary results of the present study (Supplementary Tables S3–S6). This consistency despite sample adjustment further verifies the reliability of the observed neural features of verbal fluency deficits in children with HFA.

A critical interpretative consideration is whether the observed prefrontal hypoactivation primarily reflects an executive dysfunction or could be influenced by reduced task motivation or interest in children with HFA (Li et al., 2024). As noted in recent literature, motivational factors and anhedonic traits in ASD can confound the interpretation of performance-based neural measures. While our task design ensured overt behavioral compliance and the specific correlation with social symptoms (SRS) rather than general cognition supports a deficit-based account, we cannot fully rule out the contribution of differential cognitive effort or reward valuation. Future studies incorporating subjective effort scales, physiological arousal measures, or tasks with parametrically modulated incentives would be valuable to dissociate these accounts.

Several study limitations should be acknowledged. First, aspects of the research design impose constraints on generalizability. The small sample size, along with the restriction of participants to pre-adolescent children only, may restrict generalizability to the broader HFA population, and the cross-sectional design prevents analysis of developmental changes in neural activation patterns. Additionally, the gender imbalance in our HFA sample (27 males, 2 females) reflects a well-documented challenge—females with HFA often exhibit social camouflaging of core traits, leading to underdiagnosis and recruitment difficulties (Dworzynski et al., 2012; Loomes et al., 2017)—meaning our findings are primarily representative of males and require replication in female cohorts. Second, methodological considerations warrant discussion. Although fNIRS offers excellent temporal resolution (sampling rate: 11 Hz), its spatial resolution (approximately 30 mm, referring to the minimum lateral discrimination distance) remains inferior to that of fMRI. Within the shallow probing depth of fNIRS (~1.5 cm), this resolution is adequate for detecting group-level activation in broad prefrontal areas but limits precise sub-regional localization. Future multimodal studies combining fNIRS with structural MRI would improve anatomical precision in localizing channel-specific activity. Furthermore, the current study employed a phonemic fluency task to isolate executive control aspects. Future research could directly compare phonemic and semantic fluency paradigms within the same cohort to disentangle the specific contributions of prefrontal executive systems versus temporal-lobe semantic networks to verbal fluency deficits in HFA. Despite these constraints, the robustness of the current findings is supported by consistent effect sizes across multiple prefrontal channels. Importantly, the primary between-group comparisons are based on group-level averages in a developmentally homogeneous pre-adolescent cohort, and the internal validity of these findings for the studied sample remains intact, even as the gender imbalance appropriately cautions against overgeneralization.

Three future research directions emerge from these findings. First, multi-center, large-sample longitudinal follow-up studies spanning infants, pre-adolescent and adolescent periods should characterize the developmental trajectories of prefrontal cortical activation and functional connectivity patterns in children with HFA, with targeted recruitment of females with HFA to investigate sex-specific neural patterns and validate cross-gender generalizability. Second, clinical trials could evaluate whether neuromodulation interventions targeting the IFG and FPC enhance verbal comprehension and social outcomes. Third, multimodal neuroimaging approaches integrating fNIRS with fMRI (spatial resolution: 1–2 mm) would improve anatomical precision while maintaining the superior temporal resolution of fNIRS, enabling more comprehensive network-level analyses of language processing in HFA.

## Conclusion

This fNIRS study identified significant neurofunctional differences in children with HFA during VFTs, including both attenuated prefrontal activation and reduced functional connectivity relative to TD controls. These neural measures were strongly associated with clinical profiles, demonstrating significant associations with autism symptom severity. The robust effect sizes and consistent patterns observed across multiple measures suggest that these findings may serve as reliable neural markers of impairments in the executive control of language production in HFA.

## Data Availability

The original contributions presented in the study are included in the article/Supplementary material, further inquiries can be directed to the corresponding author.

## Supplementary material

SUPPLEMENTARY FIGURE 1 Positions of the 19 fNIRS channels, and Time courses of mean Oxy-Hb concentration changes in channels during the VFT in the HFA and TD groups.
