# Supplementary figures and images for: Distinct patterns of cortical activation and functional connectivity in children with high-functioning autism during a verbal fluency task: a comparative fNIRS study
Source: Front Neurosci. 2026 Apr 1;20:1736415. doi: 10.3389/fnins.2026.1736415 (PMC13079335; doi:10.3389/fnins.2026.1736415)

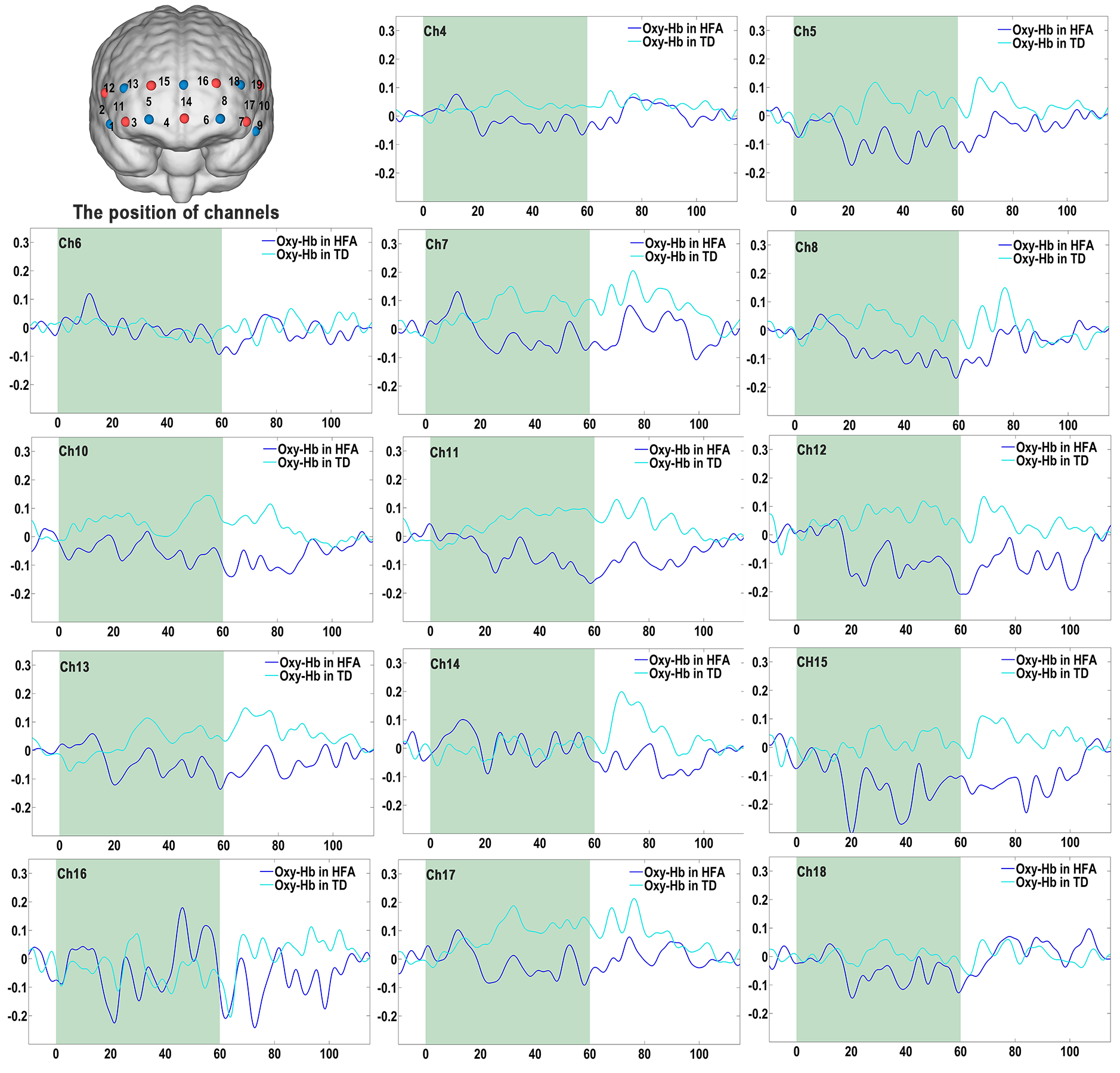

Supplement: SUPPLEMENTARY FIGURE 1 — Positions of the 19 fNIRS channels, and Time courses of mean Oxy-Hb concentration changes in channels during the VFT in the HFA and TD groups. [file Image_1.tif]
