# Supplementary material for: Distinct patterns of cortical activation and functional connectivity in children with high-functioning autism during a verbal fluency task: a comparative fNIRS study
Source: Front Neurosci. 2026 Apr 1;20:1736415. doi: 10.3389/fnins.2026.1736415 (PMC13079335; doi:10.3389/fnins.2026.1736415)
Supplement: Supplementary file 2 [file Table_1.docx]

**Supplementary Tables**

**Table S1 Comparison of channel-to-channel connection strengths between HFA and TD groups.**

| Channel to  Channel | Connectivity strength (Mean ± SD) | | *P* | FDR corrected *p* |
| --- | --- | --- | --- | --- |
|  | HFA | TD |  |  |
| 1~2 | 0.631 ± 0.319 | 0.674 ± 0.339 | 0.626 | 0.916 |
| 1~3 | 0.418 ± 0.307 | 0.501 ± 0.334 | 0.337 | 0.780 |
| 1~4 | 0.505 ± 0.326 | 0.540 ± 0.320 | 0.689 | 0.926 |
| 1~5 | 0.406 ± 0.358 | 0.526 ± 0.350 | 0.215 | 0.727 |
| 1~6 | 0.503 ± 0.342 | 0.587 ± 0.327 | 0.358 | 0.812 |
| 1~7 | 0.440 ± 0.339 | 0.462 ± 0.336 | 0.807 | 0.929 |
| 1~8 | 0.374 ± 0.366 | 0.472 ± 0.373 | 0.325 | 0.776 |
| 1~9 | 0.376 ± 0.290 | 0.492 ± 0.297 | 0.148 | 0.727 |
| 1~10 | 0.461 ± 0.332 | 0.595 ± 0.335 | 0.140 | 0.727 |
| 1~11 | 0.552 ± 0.287 | 0.577 ± 0.316 | 0.754 | 0.926 |
| 1~12 | 0.508 ± 0.336 | 0.556 ± 0.360 | 0.621 | 0.916 |
| 1~13 | 0.420 ± 0.333 | 0.502 ± 0.338 | 0.366 | 0.812 |
| 1~14 | 0.499 ± 0.336 | 0.514 ± 0.351 | 0.875 | 0.950 |
| 1~15 | 0.416 ± 0.353 | 0.587 ± 0.370 | 0.082 | 0.727 |
| 1~16 | 0.546 ± 0.324 | 0.532 ± 0.320 | 0.871 | 0.950 |
| 1~17 | 0.442 ± 0.331 | 0.502 ± 0.353 | 0.514 | 0.897 |
| 1~18 | 0.394 ± 0.333 | 0.521 ± 0.360 | 0.176 | 0.727 |
| 1~19 | 0.398 ± 0.310 | 0.505 ± 0.278 | 0.185 | 0.727 |
| 2~3 | 0.486 ± 0.292 | 0.526 ± 0.306 | 0.624 | 0.916 |
| 2~4 | 0.449 ± 0.321 | 0.558 ± 0.315 | 0.208 | 0.727 |
| 2~5 | 0.472 ± 0.332 | 0.457 ± 0.340 | 0.866 | 0.950 |
| 2~6 | 0.525 ± 0.300 | 0.428 ± 0.354 | 0.270 | 0.776 |
| 2~7 | 0.414 ± 0.331 | 0.523 ± 0.327 | 0.221 | 0.727 |
| 2~8 | 0.505 ± 0.331 | 0.457 ± 0.368 | 0.608 | 0.916 |
| 2~9 | 0.344 ± 0.286 | 0.557 ± 0.300 | 0.009 | 0.379 |
| 2~10 | 0.427 ± 0.342 | 0.560 ± 0.351 | 0.156 | 0.727 |
| 2~11 | 0.445 ± 0.355 | 0.582 ± 0.309 | 0.130 | 0.727 |
| 2~12 | 0.499 ± 0.383 | 0.616 ± 0.330 | 0.231 | 0.727 |
| 2~13 | 0.400 ± 0.351 | 0.607 ± 0.306 | 0.023 | 0.564 |
| 2~14 | 0.456 ± 0.329 | 0.401 ± 0.329 | 0.534 | 0.899 |
| 2~15 | 0.360 ± 0.347 | 0.490 ± 0.363 | 0.179 | 0.727 |
| 2~16 | 0.489 ± 0.370 | 0.429 ± 0.313 | 0.513 | 0.897 |
| 2~17 | 0.364 ± 0.323 | 0.579 ± 0.331 | 0.017 | 0.491 |
| 2~18 | 0.478 ± 0.319 | 0.586 ± 0.352 | 0.233 | 0.727 |
| 2~19 | 0.426 ± 0.323 | 0.563 ± 0.277 | 0.097 | 0.727 |
| 3~4 | 0.585 ± 0.301 | 0.605 ± 0.333 | 0.809 | 0.929 |
| 3~5 | 0.535 ± 0.320 | 0.521 ± 0.376 | 0.878 | 0.950 |
| 3~6 | 0.639 ± 0.211 | 0.523 ± 0.361 | 0.159 | 0.727 |
| 3~7 | 0.660 ± 0.262 | 0.546 ± 0.329 | 0.152 | 0.727 |
| 3~8 | 0.682 ± 0.226 | 0.453 ± 0.377 | 0.010 | 0.379 |
| 3~9 | 0.594 ± 0.288 | 0.671 ± 0.264 | 0.305 | 0.776 |
| 3~10 | 0.448 ± 0.277 | 0.503 ± 0.292 | 0.468 | 0.861 |
| 3~11 | 0.546 ± 0.339 | 0.687 ± 0.251 | 0.082 | 0.727 |
| 3~12 | 0.566 ± 0.282 | 0.590 ± 0.326 | 0.771 | 0.926 |
| 3~13 | 0.490 ± 0.311 | 0.653 ± 0.308 | 0.055 | 0.727 |
| 3~14 | 0.451 ± 0.296 | 0.590 ± 0.345 | 0.110 | 0.727 |
| 3~15 | 0.441 ± 0.334 | 0.526 ± 0.353 | 0.363 | 0.812 |
| 3~16 | 0.410 ± 0.282 | 0.446 ± 0.333 | 0.660 | 0.920 |
| 3~17 | 0.585 ± 0.307 | 0.558 ± 0.336 | 0.755 | 0.926 |
| 3~18 | 0.584 ± 0.294 | 0.521 ± 0.338 | 0.462 | 0.861 |
| 3~19 | 0.547 ± 0.336 | 0.588 ± 0.252 | 0.607 | 0.916 |
| 4~5 | 0.612 ± 0.321 | 0.513 ± 0.384 | 0.297 | 0.776 |
| 4~6 | 0.533 ± 0.322 | 0.521 ± 0.352 | 0.891 | 0.958 |
| 4~7 | 0.551 ± 0.335 | 0.590 ± 0.331 | 0.667 | 0.920 |
| 4~8 | 0.576 ± 0.330 | 0.560 ± 0.352 | 0.861 | 0.950 |
| 4~9 | 0.505 ± 0.313 | 0.505 ± 0.288 | 0.998 | 1.000 |
| 4~10 | 0.412 ± 0.323 | 0.466 ± 0.343 | 0.552 | 0.899 |
| 4~11 | 0.605 ± 0.283 | 0.524 ± 0.312 | 0.317 | 0.776 |
| 4~12 | 0.552 ± 0.326 | 0.566 ± 0.320 | 0.877 | 0.950 |
| 4~13 | 0.597 ± 0.280 | 0.570 ± 0.327 | 0.732 | 0.926 |
| 4~14 | 0.550 ± 0.308 | 0.524 ± 0.359 | 0.768 | 0.926 |
| 4~15 | 0.537 ± 0.351 | 0.537 ± 0.344 | 1.000 | 1.000 |
| 4~16 | 0.525 ± 0.313 | 0.569 ± 0.291 | 0.590 | 0.916 |
| 4~17 | 0.498 ± 0.332 | 0.539 ± 0.339 | 0.650 | 0.920 |
| 4~18 | 0.557 ± 0.306 | 0.536 ± 0.360 | 0.806 | 0.929 |
| 4~19 | 0.555 ± 0.302 | 0.533 ± 0.267 | 0.776 | 0.926 |
| 5~6 | 0.525 ± 0.342 | 0.640 ± 0.369 | 0.233 | 0.727 |
| 5~7 | 0.503 ± 0.330 | 0.563 ± 0.327 | 0.498 | 0.887 |
| 5~8 | 0.534 ± 0.344 | 0.652 ± 0.335 | 0.199 | 0.727 |
| 5~9 | 0.556 ± 0.317 | 0.557 ± 0.320 | 0.995 | 1.000 |
| 5~10 | 0.545 ± 0.287 | 0.599 ± 0.304 | 0.494 | 0.887 |
| 5~11 | 0.550 ± 0.290 | 0.645 ± 0.307 | 0.238 | 0.727 |
| 5~12 | 0.511 ± 0.346 | 0.570 ± 0.351 | 0.531 | 0.899 |
| 5~13 | 0.673 ± 0.283 | 0.600 ± 0.349 | 0.395 | 0.831 |
| 5~14 | 0.602 ± 0.295 | 0.645 ± 0.368 | 0.639 | 0.918 |
| 5~15 | 0.534 ± 0.360 | 0.672 ± 0.324 | 0.140 | 0.727 |
| 5~16 | 0.466 ± 0.328 | 0.567 ± 0.358 | 0.274 | 0.776 |
| 5~17 | 0.579 ± 0.332 | 0.586 ± 0.329 | 0.940 | 0.986 |
| 5~18 | 0.558 ± 0.336 | 0.563 ± 0.364 | 0.961 | 0.994 |
| 5~19 | 0.549 ± 0.282 | 0.533 ± 0.282 | 0.839 | 0.944 |
| 6~7 | 0.582 ± 0.288 | 0.550 ± 0.347 | 0.711 | 0.926 |
| 6~8 | 0.634 ± 0.346 | 0.655 ± 0.374 | 0.827 | 0.943 |
| 6~9 | 0.538 ± 0.287 | 0.515 ± 0.329 | 0.789 | 0.926 |
| 6~10 | 0.465 ± 0.299 | 0.548 ± 0.322 | 0.323 | 0.776 |
| 6~11 | 0.464 ± 0.330 | 0.609 ± 0.324 | 0.102 | 0.727 |
| 6~12 | 0.545 ± 0.334 | 0.475 ± 0.359 | 0.457 | 0.861 |
| 6~13 | 0.432 ± 0.341 | 0.548 ± 0.355 | 0.219 | 0.727 |
| 6~14 | 0.559 ± 0.320 | 0.687 ± 0.326 | 0.144 | 0.727 |
| 6~15 | 0.498 ± 0.368 | 0.648 ± 0.360 | 0.129 | 0.727 |
| 6~16 | 0.462 ± 0.351 | 0.604 ± 0.348 | 0.135 | 0.727 |
| 6~17 | 0.536 ± 0.313 | 0.509 ± 0.345 | 0.761 | 0.926 |
| 6~18 | 0.567 ± 0.304 | 0.533 ± 0.386 | 0.722 | 0.926 |
| 6~19 | 0.490 ± 0.266 | 0.516 ± 0.319 | 0.741 | 0.926 |
| 7~8 | 0.599 ± 0.321 | 0.560 ± 0.333 | 0.660 | 0.920 |
| 7~9 | 0.630 ± 0.257 | 0.573 ± 0.296 | 0.441 | 0.861 |
| 7~10 | 0.501 ± 0.322 | 0.568 ± 0.284 | 0.412 | 0.831 |
| 7~11 | 0.473 ± 0.322 | 0.538 ± 0.307 | 0.449 | 0.861 |
| 7~12 | 0.599 ± 0.289 | 0.538 ± 0.333 | 0.465 | 0.861 |
| 7~13 | 0.510 ± 0.341 | 0.630 ± 0.292 | 0.167 | 0.727 |
| 7~14 | 0.492 ± 0.307 | 0.448 ± 0.315 | 0.602 | 0.916 |
| 7~15 | 0.438 ± 0.328 | 0.514 ± 0.355 | 0.413 | 0.831 |
| 7~16 | 0.333 ± 0.285 | 0.482 ± 0.311 | 0.067 | 0.727 |
| 7~17 | 0.648 ± 0.274 | 0.692 ± 0.269 | 0.550 | 0.899 |
| 7~18 | 0.605 ± 0.299 | 0.608 ± 0.341 | 0.976 | 0.994 |
| 7~19 | 0.626 ± 0.289 | 0.602 ± 0.233 | 0.745 | 0.926 |
| 8~9 | 0.548 ± 0.306 | 0.460 ± 0.317 | 0.297 | 0.776 |
| 8~10 | 0.409 ± 0.330 | 0.484 ± 0.332 | 0.402 | 0.831 |
| 8~11 | 0.488 ± 0.337 | 0.514 ± 0.344 | 0.775 | 0.926 |
| 8~12 | 0.537 ± 0.335 | 0.533 ± 0.343 | 0.961 | 0.994 |
| 8~13 | 0.512 ± 0.379 | 0.616 ± 0.324 | 0.280 | 0.776 |
| 8~14 | 0.421 ± 0.377 | 0.613 ± 0.385 | 0.065 | 0.727 |
| 8~15 | 0.508 ± 0.415 | 0.646 ± 0.379 | 0.200 | 0.727 |
| 8~16 | 0.407 ± 0.336 | 0.752 ± 0.252 | 0.000 | 0.010 |
| 8~17 | 0.556 ± 0.376 | 0.505 ± 0.366 | 0.607 | 0.916 |
| 8~18 | 0.667 ± 0.316 | 0.724 ± 0.310 | 0.498 | 0.887 |
| 8~19 | 0.503 ± 0.312 | 0.499 ± 0.329 | 0.965 | 0.994 |
| 9~10 | 0.581 ± 0.282 | 0.634 ± 0.253 | 0.465 | 0.861 |
| 9~11 | 0.536 ± 0.309 | 0.667 ± 0.260 | 0.094 | 0.727 |
| 9~12 | 0.464 ± 0.302 | 0.637 ± 0.280 | 0.032 | 0.676 |
| 9~13 | 0.501 ± 0.306 | 0.693 ± 0.237 | 0.011 | 0.379 |
| 9~14 | 0.451 ± 0.294 | 0.543 ± 0.294 | 0.233 | 0.727 |
| 9~15 | 0.402 ± 0.335 | 0.522 ± 0.299 | 0.166 | 0.727 |
| 9~16 | 0.329 ± 0.273 | 0.424 ± 0.284 | 0.206 | 0.727 |
| 9~17 | 0.699 ± 0.267 | 0.620 ± 0.301 | 0.298 | 0.776 |
| 9~18 | 0.530 ± 0.319 | 0.571 ± 0.277 | 0.615 | 0.916 |
| 9~19 | 0.620 ± 0.307 | 0.744 ± 0.215 | 0.085 | 0.727 |
| 10~11 | 0.501 ± 0.317 | 0.618 ± 0.286 | 0.155 | 0.727 |
| 10~12 | 0.450 ± 0.338 | 0.552 ± 0.326 | 0.258 | 0.773 |
| 10~13 | 0.548 ± 0.307 | 0.617 ± 0.282 | 0.385 | 0.831 |
| 10~14 | 0.556 ± 0.289 | 0.521 ± 0.313 | 0.667 | 0.920 |
| 10~15 | 0.534 ± 0.312 | 0.568 ± 0.305 | 0.677 | 0.926 |
| 10~16 | 0.493 ± 0.333 | 0.464 ± 0.311 | 0.743 | 0.926 |
| 10~17 | 0.483 ± 0.307 | 0.489 ± 0.316 | 0.937 | 0.986 |
| 10~18 | 0.467 ± 0.290 | 0.493 ± 0.336 | 0.753 | 0.926 |
| 10~19 | 0.533 ± 0.299 | 0.663 ± 0.233 | 0.077 | 0.727 |
| 11~12 | 0.576 ± 0.318 | 0.609 ± 0.323 | 0.703 | 0.926 |
| 11~13 | 0.625 ± 0.321 | 0.654 ± 0.319 | 0.738 | 0.926 |
| 11~14 | 0.490 ± 0.329 | 0.669 ± 0.314 | 0.042 | 0.727 |
| 11~15 | 0.492 ± 0.323 | 0.595 ± 0.319 | 0.237 | 0.727 |
| 11~16 | 0.454 ± 0.316 | 0.543 ± 0.334 | 0.311 | 0.776 |
| 11~17 | 0.504 ± 0.320 | 0.578 ± 0.334 | 0.398 | 0.831 |
| 11~18 | 0.451 ± 0.344 | 0.520 ± 0.353 | 0.458 | 0.861 |
| 11~19 | 0.529 ± 0.295 | 0.609 ± 0.276 | 0.301 | 0.776 |
| 12~13 | 0.488 ± 0.330 | 0.716 ± 0.244 | 0.005 | 0.379 |
| 12~14 | 0.509 ± 0.345 | 0.519 ± 0.332 | 0.909 | 0.966 |
| 12~15 | 0.506 ± 0.366 | 0.532 ± 0.348 | 0.790 | 0.926 |
| 12~16 | 0.463 ± 0.362 | 0.553 ± 0.322 | 0.331 | 0.776 |
| 12~17 | 0.491 ± 0.338 | 0.545 ± 0.313 | 0.542 | 0.899 |
| 12~18 | 0.587 ± 0.313 | 0.537 ± 0.349 | 0.572 | 0.916 |
| 12~19 | 0.464 ± 0.311 | 0.557 ± 0.248 | 0.227 | 0.727 |
| 13~14 | 0.562 ± 0.299 | 0.565 ± 0.362 | 0.977 | 0.994 |
| 13~15 | 0.574 ± 0.344 | 0.594 ± 0.342 | 0.834 | 0.944 |
| 13~16 | 0.472 ± 0.317 | 0.582 ± 0.295 | 0.185 | 0.727 |
| 13~17 | 0.486 ± 0.337 | 0.606 ± 0.327 | 0.184 | 0.727 |
| 13~18 | 0.564 ± 0.314 | 0.690 ± 0.305 | 0.133 | 0.727 |
| 13~19 | 0.577 ± 0.272 | 0.654 ± 0.237 | 0.265 | 0.776 |
| 14~15 | 0.672 ± 0.310 | 0.739 ± 0.264 | 0.390 | 0.831 |
| 14~16 | 0.546 ± 0.349 | 0.606 ± 0.377 | 0.536 | 0.899 |
| 14~17 | 0.442 ± 0.324 | 0.493 ± 0.351 | 0.579 | 0.916 |
| 14~18 | 0.584 ± 0.318 | 0.573 ± 0.351 | 0.905 | 0.966 |
| 14~19 | 0.502 ± 0.310 | 0.552 ± 0.299 | 0.544 | 0.899 |
| 15~16 | 0.614 ± 0.305 | 0.590 ± 0.350 | 0.791 | 0.926 |
| 15~17 | 0.400 ± 0.366 | 0.531 ± 0.358 | 0.181 | 0.727 |
| 15~18 | 0.638 ± 0.353 | 0.674 ± 0.335 | 0.696 | 0.926 |
| 15~19 | 0.467 ± 0.326 | 0.616 ± 0.256 | 0.066 | 0.727 |
| 16~17 | 0.364 ± 0.296 | 0.449 ± 0.351 | 0.331 | 0.776 |
| 16~18 | 0.511 ± 0.316 | 0.629 ± 0.330 | 0.175 | 0.727 |
| 16~19 | 0.439 ± 0.311 | 0.478 ± 0.294 | 0.633 | 0.917 |
| 17~18 | 0.506 ± 0.350 | 0.588 ± 0.380 | 0.404 | 0.831 |
| 17~19 | 0.639 ± 0.262 | 0.674 ± 0.251 | 0.611 | 0.916 |
| 18~19 | 0.548 ± 0.325 | 0.635 ± 0.283 | 0.294 | 0.776 |

**Table S2 Associations between channel Oxy-Hb changes and demographic characteristics in the HFA group.**

| Channel names | Age | | Being an  only child  (Yes/No) | | Parental care  (Yes/No) | | Gender (Male/Female) | | Natural birth (yes/no) | |
| --- | --- | --- | --- | --- | --- | --- | --- | --- | --- | --- |
|  | *r* | *p* | *r_s_* | *p* | *r_s_* | *p* | *r_s_* | *p* | *r_s_* | *p* |
| Ch1 | 0.036 | 0.845 | -0.184 | 0.339 | 0.231 | 0.228 | -0.033 | 0.867 | -0.034 | 0.861 |
| Ch2 | -0.296 | 0.119 | -0.033 | 0.863 | 0.033 | 0.865 | 0.114 | 0.556 | -0.212 | 0.269 |
| Ch3 | 0.365 | 0.052 | 0.109 | 0.574 | 0.091 | 0.640 | -0.277 | 0.146 | -0.042 | 0.827 |
| Ch4 | 0.199 | 0.300 | -0.042 | 0.829 | 0.099 | 0.609 | 0.081 | 0.675 | -0.008 | 0.965 |
| Ch5 | 0.172 | 0.372 | 0.335 | 0.076 | 0.157 | 0.417 | -0.033 | 0.867 | -0.212 | 0.269 |
| Ch6 | 0.075 | 0.698 | -0.184 | 0.339 | 0.157 | 0.417 | -0.114 | 0.556 | 0.119 | 0.539 |
| Ch7 | -0.002 | 0.990 | 0.176 | 0.362 | -0.008 | 0.966 | 0.033 | 0.867 | -0.221 | 0.250 |
| Ch8 | 0.366 | 0.051 | 0.100 | 0.604 | 0.115 | 0.551 | 0.114 | 0.556 | 0.204 | 0.289 |
| Ch9 | 0.196 | 0.307 | 0.268 | 0.160 | -0.140 | 0.468 | 0.130 | 0.501 | -0.272 | 0.154 |
| Ch10 | 0.090 | 0.642 | -0.151 | 0.435 | 0.058 | 0.766 | 0.277 | 0.146 | -0.034 | 0.861 |
| Ch11 | 0.018 | 0.928 | -0.360 | 0.055 | 0.107 | 0.580 | -0.016 | 0.933 | -0.178 | 0.355 |
| Ch12 | -0.203 | 0.292 | -0.310 | 0.102 | 0.099 | 0.609 | -0.211 | 0.271 | -0.017 | 0.930 |
| Ch13 | -0.122 | 0.528 | -0.017 | 0.931 | 0.091 | 0.640 | 0.000 | 1.000 | 0.008 | 0.965 |
| Ch14 | 0.171 | 0.376 | -0.134 | 0.489 | 0.115 | 0.551 | 0.065 | 0.737 | 0.008 | 0.965 |
| Ch15 | 0.227 | 0.236 | 0.017 | 0.931 | 0.140 | 0.468 | 0.114 | 0.556 | -0.229 | 0.231 |
| Ch16 | 0.046 | 0.812 | -0.410 | 0.027 | 0.247 | 0.196 | 0.130 | 0.501 | -0.178 | 0.355 |
| Ch17 | 0.057 | 0.769 | 0.033 | 0.863 | -0.165 | 0.392 | 0.081 | 0.675 | -0.289 | 0.129 |
| Ch18 | 0.293 | 0.123 | -0.117 | 0.545 | -0.049 | 0.799 | -0.049 | 0.802 | 0.025 | 0.896 |
| Ch19 | 0.275 | 0.149 | -0.042 | 0.829 | 0.140 | 0.468 | 0.244 | 0.202 | -0.195 | 0.310 |

**Table S3 Demographic and clinical characteristics (mean ± SD).**

|  | **HFA** **(6-13 *y*)**  **(n=29)** | **TD (6-13 *y*)**  **(n=23)** | ***t* / Chi-square value** | ***p*** |
| --- | --- | --- | --- | --- |
| ***Demographics*** | | | | |
| Age (years) | 7.69±1.83 | 8.39±1.41 | -1.514 | 0.136 |
| Gender (male/female) | 28/1 | 20/3 | 1.663 | 0.197 |
| delivery mode (yes/no) | 18/11 | 11/12 | 1.005 | 0.304 |
| Parental care (yes/no) | 16/13 | 16/7 | 1.123 | 0.289 |
| Being an only child (yes/no) | 17/12 | 16/7 | 0.663 | 0.416 |
| ***Clinical characteristics*** | | | | |
| Wechsler Children's Intelligence Test-IV | | | | |
| Verbal comprehension Index (VCI) | 72.79±18.81 | 86.91±15.72 | -2.886 | **0.006** |
| Perceptual reasoning Index (PRI) | 96.97±17.91 | 94.22±18.49 | 0.542 | 0.590 |
| Full-Scale Intelligence Quotient (FSIQ) | 80.76±12.10 | 87.30±16.94 | -1.625 | 0.111 |
| Social Responsiveness Scale (SRS) | 94.10±17.50 | 34.48±6.25 | 15.544 | **<0.001** |
| ***VFT Behavioral Performance*** |  |  |  |  |
| Correct Words / Minute | 3.31±1.17 | 3.78±0.95 | -1.569 | 0.123 |

**Table S4 Differences in oxygenated hemoglobin concentration during two sets of channel-based task states.**

| **Channel** | **S-D** | **HFA**  **(6-13 *y*)** | **TD**  **(6-13 *y*)** | ***t*** | ***FDR-corrected p*** |
| --- | --- | --- | --- | --- | --- |
| 1 | S2-D2 | -0.0379 | 0.2189 | -3.180 | **0.012** |
| 2 | S2-D7 | -0.1364 | 0.1133 | -4.609 | **0.001** |
| 3 | S3-D2 | -0.0210 | 0.0970 | -3.326 | **0.010** |
| 4 | S3-D3 | -0.0070 | 0.0407 | -1.131 | 0.334 |
| 5 | S3-D8 | -0.0840 | 0.0324 | -2.046 | 0.109 |
| 6 | S4-D3 | 0.0067 | 0.0138 | -0.121 | 0.904 |
| 7 | S4-D4 | -0.0069 | 0.0832 | -1.808 | 0.145 |
| 8 | S4-D9 | -0.0667 | 0.0223 | -1.957 | 0.118 |
| 9 | S5-D4 | -0.0002 | 0.1551 | -3.022 | **0.015** |
| 10 | S5-D10 | -0.0384 | 0.0546 | -1.709 | 0.162 |
| 11 | S8-D2 | -0.0396 | 0.0683 | -2.260 | 0.076 |
| 12 | S8-D7 | -0.0210 | 0.0538 | -1.503 | 0.203 |
| 13 | S8-D8 | -0.0239 | 0.0275 | -1.006 | 0.379 |
| 14 | S9-D3 | 0.0153 | -0.0064 | 0.510 | 0.684 |
| 15 | S9-D8 | -0.1202 | 0.0082 | -1.549 | 0.202 |
| 16 | S9-D9 | -00225 | -0.0470 | 0.283 | 0.821 |
| 17 | S10-D4 | -0.0164 | 0.0943 | -2.425 | 0.060 |
| 18 | S10-D9 | -0.0496 | 0.0128 | -1.385 | 0.234 |
| 19 | S10-D10 | -0.0591 | 0.1097 | -3.378 | **0.010** |

**Table S5 Comparison of mean channel-to-channel connectivity strength between HFA (6-13 y) and TD (6-13 y) groups.**

| **Group** | **n** | **Mean connectivity strength (Mean ± SD)** | ***p*** |
| --- | --- | --- | --- |
| HFA | 29 | 0.485 ± 0.220 | < 0.001 |
| TD | 23 | 0.595 ± 0.279 |  |

**Table S6** **Associations between channel Oxy-Hb changes and clinical characteristics in the HFA (6-13 *y*) group.**

| Channel | Verbal comprehension Index (VCI) | | Perceptual reasoning Index (PRI) | | Full-Scale Intelligence Quotient (FSIQ) | | Social Responsiveness Scale (SRS) | |
| --- | --- | --- | --- | --- | --- | --- | --- | --- |
|  | *r* | *p* | *r* | *p* | *r* | *p* | *r* | *p* |
| 1 | -0.051 | 0.792 | -0.208 | 0.280 | -0.257 | 0.178 | -0.524 | **<0.001** |
| 2 | 0.131 | 0.498 | -0.060 | 0.756 | -0.023 | 0.904 | -0.586 | **<0.001** |
| 3 | -0.096 | 0.621 | 0.201 | 0.297 | 0.015 | 0.938 | -0.385 | **0.039** |
| 4 | -0.188 | 0.328 | 0.359 | 0.056 | 0.074 | 0.701 | -0.151 | 0.435 |
| 5 | 0.090 | 0.641 | -0.135 | 0.486 | -0.090 | 0.641 | -0.254 | 0.185 |
| 6 | -0.270 | 0.156 | 0.240 | 0.210 | -0.079 | 0.685 | 0.016 | 0.936 |
| 7 | -0.160 | 0.408 | 0.071 | 0.715 | -0.163 | 0.397 | -0.167 | 0.387 |
| 8 | -0.184 | 0.341 | 0.110 | 0.569 | -0.001 | 0.996 | -0.040 | 0.835 |
| 9 | 0.206 | 0.283 | -0.101 | 0.603 | -0.051 | 0.791 | -0.108 | 0.578 |
| 10 | 0.039 | 0.840 | -0.042 | 0.829 | -0.015 | 0.937 | -0.040 | 0.839 |
| 11 | 0.244 | 0.202 | -0.194 | 0.313 | -0.060 | 0.756 | -0.125 | 0.517 |
| 12 | -0.156 | 0.418 | 0.014 | 0.944 | -0.263 | 0.167 | -0.199 | 0.301 |
| 13 | -0.017 | 0.929 | -0.148 | 0.443 | -0.246 | 0.199 | -0.334 | 0.076 |
| 14 | 0.184 | 0.340 | 0.085 | 0.661 | 0.102 | 0.600 | 0.064 | 0.741 |
| 15 | 0.203 | 0.290 | -0.147 | 0.448 | 0.009 | 0.964 | 0.068 | 0.725 |
| 16 | 0.263 | 0.168 | -0.256 | 0.180 | -0.056 | 0.771 | 0.091 | 0.637 |
| 17 | -0.047 | 0.808 | -0.141 | 0.465 | -0.259 | 0.175 | -0.073 | 0.706 |
| 18 | 0.014 | 0.941 | -0.011 | 0.954 | -0.019 | 0.923 | 0.092 | 0.635 |
| 19 | 0.043 | 0.826 | 0.066 | 0.733 | 0.022 | 0.910 | -0.039 | 0.840 |
